# Repurposing electroencephalogram monitoring of general anaesthesia for building biomarkers of brain ageing: an exploratory study

**DOI:** 10.1016/j.bjao.2023.100145

**Published:** 2023-06-16

**Authors:** David Sabbagh, Jérôme Cartailler, Cyril Touchard, Jona Joachim, Alexandre Mebazaa, Fabrice Vallée, Étienne Gayat, Alexandre Gramfort, Denis A. Engemann

**Affiliations:** 1 INSERM, Université de Paris, Paris, France; 2 Inria, CEA, Université Paris-Saclay, Palaiseau, France; 3 Department of Anesthesia and Critical Care Medicine, AP-HP, Hôpital Lariboisière, Paris, France; 4 Department of Neurology, Max Planck Institute for Human Cognitive and Brain Sciences, Leipzig, Germany; 5 Roche Pharma Research and Early Development, Neuroscience and Rare Diseases, Roche Innovation Center Basel, F. Hoffmann-La Roche Ltd, Basel, Switzerland

**Keywords:** brain age, burst suppression, electroencephalogram (EEG), general anaesthesia, machine learning, propofol, sevoflurane

## Abstract

**Background:**

Electroencephalography (EEG) is increasingly used for monitoring the depth of general anaesthesia, but EEG data from general anaesthesia monitoring are rarely reused for research. Here, we explored repurposing EEG monitoring from general anaesthesia for brain-age modelling using machine learning. We hypothesised that brain age estimated from EEG during general anaesthesia is associated with perioperative risk.

**Methods:**

We reanalysed four-electrode EEGs of 323 patients under stable propofol or sevoflurane anaesthesia to study four EEG signatures (95% of EEG power <8–13 Hz) for age prediction: total power, alpha-band power (8–13 Hz), power spectrum, and spatial patterns in frequency bands. We constructed age-prediction models from EEGs of a healthy reference group (ASA 1 or 2) during propofol anaesthesia. Although all signatures were informative, state-of-the-art age-prediction performance was unlocked by parsing spatial patterns across electrodes along the entire power spectrum (mean absolute error=8.2 yr; *R*^2^=0.65).

**Results:**

Clinical exploration in ASA 1 or 2 patients revealed that brain age was positively correlated with intraoperative burst suppression, a risk factor for general anaesthesia complications. Surprisingly, brain age was negatively correlated with burst suppression in patients with higher ASA scores, suggesting hidden confounders. Secondary analyses revealed that age-related EEG signatures were specific to propofol anaesthesia, reflected by limited model generalisation to anaesthesia maintained with sevoflurane.

**Conclusions:**

Although EEG from general anaesthesia may enable state-of-the-art age prediction, differences between anaesthetic drugs can impact the effectiveness and validity of brain-age models. To unleash the dormant potential of EEG monitoring for clinical research, larger datasets from heterogeneous populations with precisely documented drug dosage will be essential.

Electroencephalography (EEG) is often used during general anaesthesia for monitoring anaesthetic depth and adjusting drug dosage.^1^, ^2^, ^3^, ^4^, ^5^ The increasing availability of EEG recordings from general anaesthesia has stimulated cross-cutting research linking anaesthesiology and neuroscience.^6^, ^7^, ^8^ EEG dynamics during general anaesthesia and the drug dosage required for achieving stable anaesthesia can depend on age and general health.^9^ Cognitive decline has been associated with reduced drug requirement and EEG power changes in the alpha frequency band (8–13 Hz).^10^^,^^11^ These recent results raise the possibility that brain health may be reflected in EEG signals observed during general anaesthesia.^12^

It follows that EEG during general anaesthesia might be used as a screening tool to assess the risk of developing neurodegenerative diseases. Several factors are favourable to this idea. First, EEG signals during stable anaesthesia can be of high signal quality, as neuromuscular block minimises movement artifacts. Second, the availability of EEG recordings from general anaesthesia keeps growing, as general anaesthesia is conducted worldwide in all age groups, potentially avoiding selection bias, which may be a factor in laboratory research.^13^^,^^14^ Third, recent progress in advanced analytics and machine learning for EEG has led to the development of novel biomarkers of cognition, brain function, and health.^15^, ^16^, ^17^ When combined, these factors could turn EEG monitoring data obtained during general anaesthesia into a valuable tool for clinical research.

In this study, we explore the possibility of repurposing existing EEG monitoring data from general anaesthesia for building data-driven measures of brain ageing.^18^ Specifically, we propose to translate the previously developed brain-age framework^19^ to the context of general anaesthesia. By using machine learning, brain age can capture individual ageing. This framework provides quantitative comparisons of the chronological age of a person against their statistically expected age given their brain data (‘this brain looks older/younger’).^20^^,^^21^ Over recent years, numerous studies^19^^,^^20^^,^^22^^,^^23^ have found that brain-age estimates from the general population yield sensitive measures of neurodegenerative risk and disease severity in clinical populations.^24^

The bulk of the brain-age literature is based on MRI,^23^^,^^25^, ^26^, ^27^ and brain age is believed to mainly reflect cortical atrophy. Although MRI-based brain age shows leading state-of-the-art performance (age prediction with 2–5 yr mean absolute error [MAE]), the present focus on MRI hampers exploration of brain age in situations in which MRI is not available. A promising line of research has demonstrated that brain age can be estimated from EEG (current state-of-the-art age prediction with around 8 yr MAE) and can even contribute additional information to MRI^20^^,^^28^^,^^29^ by providing insights into neuronal activity.

Applied to general anaesthesia, EEG-derived brain age could help develop a real-time intraoperative biomarker of brain health, facilitating individualised treatment. Brain age estimated during an intervention could also be used as a postoperative indicator to systematically assess the risk of abnormal cognition and identify patients who may benefit from a consultation with a neurologist (e.g. a memory consultation).

It is currently unknown whether brain age can be estimated from EEG during general anaesthesia. Most EEG approaches for brain-age modelling have relied on fine-grained spatial information provided by dozens to hundreds of electrodes and data collected in the laboratory setting.^17^^,^^28^ However, brain age has recently been estimated from sleep EEG with fewer electrodes.^18^^,^^30^ Another source of potential complications for estimating brain age from EEG during general anaesthesia is related to the type of anaesthetic drug. For example, compared with propofol anaesthesia, inhalation anaesthesia can shape EEG activity differently, increasing modelling complexity. Moreover, the amount of anaesthetic drug given to a patient to achieve stable anaesthesia may by itself reflect the patient's cognitive health,^10^ potentially inducing confounding effects.

Our primary objective was to demonstrate the feasibility of modelling chronological age as a function of EEG power from intraoperative recordings in ASA 1 or 2 patients for whom the perioperative risk should be relatively low. We used state-of-the-art machine learning methods to estimate model prediction performance and hypothesised that the entire power spectrum was informative rather than specific frequency bands commonly used for monitoring anaesthetic depth. Our secondary objective was to investigate the association between chronological age, EEG-derived brain age, and intraoperative burst suppression in patients with ASA scores of 1 or 2 and those with higher scores (ASA 3) and hence higher perioperative risk. Finally, we investigated the specificity of the propofol-induced EEG signature of brain age through generalisation testing against EEG collected under sevoflurane anaesthesia.

## Methods

We reanalysed EEG and clinical data from the Brain Power Spectral Density Under Propofol (PROBRAIN) study registered under the ID NCT03876379 and approved by the Société de Réanimation de Langue Française (SRLF; Paris, France) Ethics Advisory Committee (chairperson: Dr Jean Reignier) on 5 January 2016, under the reference CE SRLF 11–356. SRLF is the French national academic society for anaesthesia and critical care consulted by the Department of Anaesthesiology at the Lariboisière Hospital (Paris, France). Patients were provided with an information letter. Verbal consent was recorded from every patient before anaesthesia.

### Patient selection

**Fig. 1:**
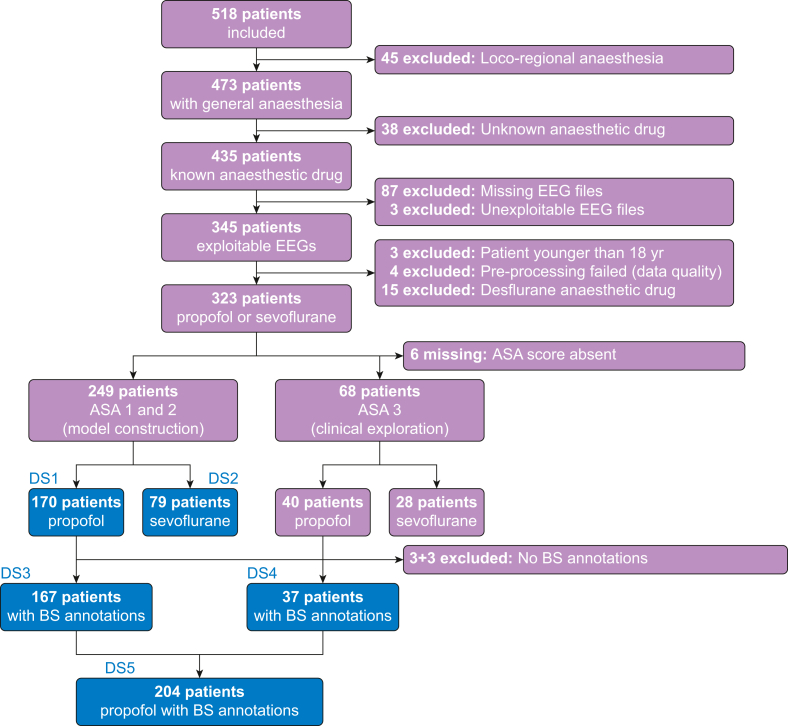
Flowchart illustrating patient selection and distinct data subsets used for data analysis. BS, burst suppression.

Between September 2017 and January 2020, patients undergoing an elective interventional procedure under general anaesthesia (for orthopaedic surgery or neuroradiology intervention for asymptomatic aneurysm) were selected to participate in the prospective, observational, monocentric PROBRAIN study at the Lariboisière Hospital. This sample was built by opportunistically including all EEG monitoring data available and compatible with the data collection procedure. The following exclusion criteria applied: pregnant women, age below 18 yr, patients receiving sedation and mechanical ventilation of the lungs before the procedure, history of bleeding aneurysm, neurodegenerative disease, neurological disorders, and untreated depression. The patient selection is illustrated with a flowchart (Fig. 1).

### Anaesthetic protocol

General anaesthesia was administered according to standard practices. An opioid was administered (sufentanil 0.2 μg kg^−1^ h^−1^ for orthopaedic patients and remifentanil 3–5 ng ml^−1^ for neuroradiology patients) followed by i.v. induction of anaesthesia using propofol. Atracurium was used for neuromuscular block, and anaesthesia was maintained using propofol or sevoflurane at the discretion of the anaesthesiologist in charge. Propofol was administered using a target-controlled infusion (TCI) system with a brain effect-site concentration ranging from 3 to 3.5 μg ml^−1^ according to the Schnider model,^31^ whereas the end-tidal sevoflurane concentration was typically 1.5–2%. Propofol and sevoflurane doses were adjusted to maintain a spectral edge frequency 95 (SEF95) between 8 and 13 Hz during anaesthetic maintenance. For comparability, we used this frequency range as the definition of stable anaesthesia for all analyses in this study. The MAP during the entire intervention was maintained at 90% of its reference value and always above 65 mm Hg.

### Data collection

Cerebral activity during general anaesthesia was monitored using a Masimo™ SedLine® device with a four-frontal electrode EEG montage (Fp1, Fp2, F7, and F8, referenced on Cz), sampled at 63 Hz by default. EEG sub-hairline electrodes were placed a few minutes before general anaesthesia induction and removed shortly after recovery of consciousness. Intraoperative EEG data were then extracted from the device, anonymised, cleaned from burst suppression and artifacts, and stored on a file server in enhanced disk format (EDF). In other words, EEG segments containing burst suppression were never used for model construction. We retrieved patient characteristics (age, gender, weight, height, and BMI) and clinical information (type of anaesthetic drug and ASA score) from the anaesthetic assessment consultation.

### EEG processing, model construction, and clinical exploration

**Fig. 2:**
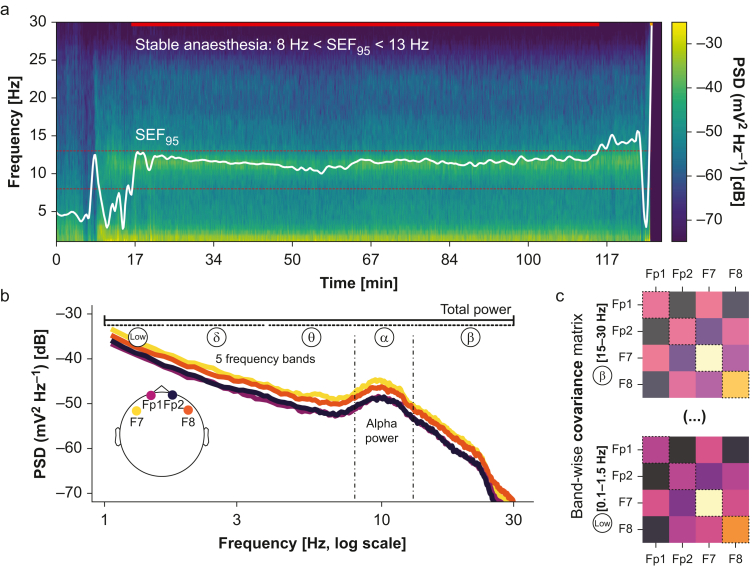
Building biomarkers of brain ageing from stable segments of general anaesthesia. (a) We selected the longest consecutive period of stable anaesthesia (propofol), defined by the spectral edge frequency 95 (SEF95) falling in the 8–13 Hz range and the absence of high-voltage artifacts or burst suppression. (b) We estimated the power spectral density (PSD), which can reveal EEG signatures commonly used in anaesthetic monitoring, such as total power or alpha power. Furthermore, we investigated the hypothesis that additional information on targets beyond clinical monitoring (i.e. brain age is distributed across the entire power spectrum). For this purpose, we adaptively summarised the PSD over all frequencies and channels using supervised learning. Reference models for estimating brain age from EEG have exploited spatial patterns in different frequency bands presented by the covariance between EEG channels (c) whose diagonal represents the PSD in the given frequency band. To assess the complementarity of these readouts, we used the stacking approach in which (i) ‘alpha power’, (ii) ‘total power’, (iii) the ‘power spectrum’, and (iv) ‘spatial patterns’ from five frequency bands were gradually included. Model comparisons established the relative merit of these increasingly complex features.

The EEG processing and feature extraction is outlined in Figure 2. In summary, the signal from each patient was divided into epochs using 60-s sliding windows with a 10-s shift. For every epoch, we computed two different types of power spectral features. First, we estimated the power spectral density (PSD) between 0 and 30 Hz using Welch's method. Second, we computed the 4 × 4 covariance matrices between all four electrodes, in five frequency bands, leading to five 4 × 4 matrices per epoch. Each of these five matrices contains the corresponding band powers of the EEG signal of a particular electrode in its diagonal and the cross-powers between electrodes in its off-diagonal terms. Compared with PSD, the covariance matrices encapsulate additional information about the spatial distribution of the power spectrum. After discarding epochs with peak-to-peak amplitude below 0.1 μV on any of the four electrodes (to avoid inclusion of artifacts or burst suppression), we retained the longest period of consecutive epochs (during anaesthesia maintenance) for which the average SEF95 across the four channels falls in the 8–13 Hz range. The PSDs and the covariance matrices were then averaged across the selected epochs. All features were computed using the open-source software package coffeine (https://github.com/coffeine-labs/coffeine), which implements and documents the methods from our previous work.

We applied brain-age models and methods for model comparisons from previous reference publications.^20^^,^^28^ We performed linear regression (Ridge regression) based on four different sets of features, each model complementing the previous one by including additional EEG features. First, the ‘total power’ model summed EEG power over the whole range (1–30 Hz), averaged across all four electrodes (one feature), representing the hypothesis of spectrally uniform information. Second, the ‘alpha power’ model added EEG power averaged between 8 and 13 Hz across all four electrodes (one additional feature; two features total), representing the hypothesis of a local spectral effect in the frequency range used for monitoring anaesthetic depth. Third, the ‘power spectrum’ model added the PSD at uniformly spaced frequencies between 1 and 30 Hz averaged across all four electrodes (16 features), representing the hypothesis of distributed spectral effects. Finally, the ‘spatial patterns’ model used the 4 × 4 covariances in five frequency bands (low [0.1–1.5 Hz], delta [1.5–4 Hz], theta [4–8 Hz], alpha [8–15 Hz], and beta [15–30 Hz]), representing the hypothesis of distributed spatio-spectral effects (50 features). The first three models used only the PSD, and the last used the covariance matrices.

By performing model comparisons, we can understand the relative merit of these increasingly complex features and assess their complementarity. For statistically balanced comparisons between models based on different numbers of features, the stacking method was used as in previous brain-age publications.^20^^,^^22^ This approach integrates the different sets of features through a second-level model, which gets one input per feature set. The prediction performance between the different stacking models preserves the performance of the simpler model. Hence, improved performance can only be attributed to additional information while reducing the risk of overfitting by representing every feature set with one single input (first-level model prediction). Ridge regression was used for both the first-level and second-level prediction algorithms with 100 (Monte Carlo) cross-validation (CV) splits. This design choice uses stacking simply to decorrelate the inputs and represent every feature set by one component while not adding any non-linearity.

To date, no generally accepted procedure exists for defining statistically justified null-hypothesis significance tests of observed performance rankings between pairs of prediction models. However, the CV distribution itself provides valuable uncertainty estimates. To gauge practical significance, we followed the strategy from previous work^20^^,^^28^ and plotted or reported percentiles of the CV distribution (*P*_2.5_, *P*_25_, *P*_75_, and *P*_97.5_) alongside rank statistics counting on how many splits one model was better than the other. Model superiority was then assessed via pair-wise ranking across CV splits.^20^^,^^28^ Chance level was assessed by a dummy regressor predicting the mean outcome of the training data for each sample. For model construction, we focused on patients with low ASA scores under propofol anaesthesia (ASA 1 or 2; *n*=170; see DS1 in Fig. 1). The brain ages of these healthy reference subjects should match their chronological ages. This allows us to estimate a reference model of normally expected ageing-related EEG patterns by calibrating and validating it using CV on the same set of patients with ASA score of 1 or 2. To investigate the clinical utility of the reference model for anomaly detection, we then studied the potential drift and deviation in model predictions from the reference population by applying the model to patient populations with higher ASA scores (second analysis). As an exploratory endpoint, we compared brain age with burst suppression (a common predictor of postoperative complications). We computed cross-validated brain-age predictions^20^ on the data used for model building (ASA 1 or 2; DS3 in Fig. 1) and then predicted brain age in a dataset from a population with higher ASA scores that was not used for model building (ASA 3; DS4 in Fig. 1). The brain-age variable was then obtained by concatenating the predictions across the two datasets (DS5 in Fig. 1). For additional details on modelling, definition of burst suppression, and details on statistical analyses and software, see the supplementary material. We hypothesised that our model calibrated on patients with lower ASA scores would make informative prediction errors on the population with higher ASA scores, and that these errors would be associated with an increase in burst suppression.

## Results

**Fig. 3:**
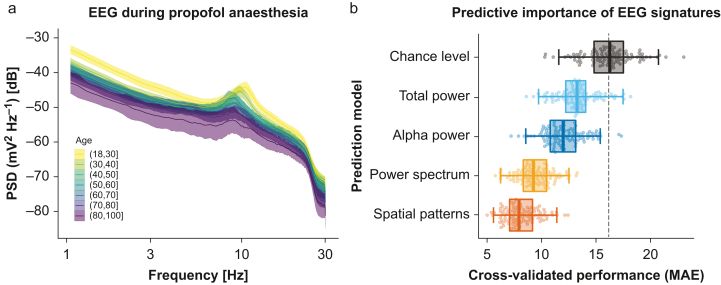
Modelling brain ageing from spatio-spectral EEG signatures during propofol anaesthesia. (a) Average power spectral density (PSD) by age group represented by different colours (*n*=170; ASA 1 or 2). Shaded areas represent twice the standard error of the mean for every age group. Power and peak frequencies tend to decrease with age. (b) Cross-validated performance of age-prediction models based on progressive inclusion of different EEG signatures (Monte Carlo; 100 splits). Dots represent scores at individual cross-validation splits, box plots summarise the cross-validation distribution, and whiskers show the 2.5th and 97.5th percentiles. The dotted line indicates the expected performance for a chance-level model. Results are ordered by prediction error, descending from top to bottom. Models include EEG signatures from previous models (e.g. ‘alpha power’ refers to combining total power and alpha power). Informal analyses revealed that spatial patterns enabled virtually identical performance as the final model combining the diverse EEG signatures. For simplicity, we focused on the spatial patterns model in all subsequent analysis. MAE, mean absolute error.

We considered 323 patients (see Table 1 for details) with both a properly concatenated EEG recording and metadata information. Analysis-defining subsets of patients are listed in Figure 1. We first explored the relationship between EEG activity and ageing across the frequency spectrum under propofol anaesthesia (Fig. 3), which was the more common maintenance anaesthetic administered in this study (Table 1; Fig. 1, DS1). Binning the power spectra by age groups revealed age-related patterns (Fig. 3a). Across frequencies, younger patients tended to show higher EEG power. We formally quantified this tendency using a linear mixed effects model regressing the log power (dB) on age, log frequency, and their interaction (intercepts varying by patient). The analysis uncovered that regardless of frequency, EEG power declined on average by –0.10 dB; 95% confidence interval (CI) [–0.13 to –0.08] with every year of age. The full model is summarised in Supplementary Table S1.

**Table 1:** Patient characteristics. Data are presented as median [25th–75th percentiles].

Variable	Number of patients or median
Female/male	211/112
Age (yr)	57 [39–70]
Body weight (kg)	74 [61–85]
BMI (kg m^−2^)	25 [22–29]
Propofol/sevoflurane	216/107
ASA 1 or 2/ASA 3	249/68

Figure 3b presents model comparisons for combinations of different EEG signatures. Chance level was estimated around 16 yr of MAE (*P*_25_=14.8; *P*_75_=17.5). The ‘total power’ model led to an improvement of the CV score by about –2.9 yr MAE on average (*P*_25_=–4.12; *P*_75_=–1.9), performing better than chance in 95/100 CV splits. As average alpha power was included, the CV scores improved by another –1.2 yr MAE (*P*_25_=–1.9; *P*_75_=–0.34), improving on the previous model on 86/100 CV splits. Adding fine-grained spectral information across all frequencies from 1 to 30 Hz led to further improvements by –2.7 yr MAE (*P*_25_=–3.7; *P*_75_=–1.86), outperforming the previous model on 95/100 CV splits. Finally, including information about the spatial patterns of covariance between the electrodes lowered the CV score by another –1.22 yr MAE (*P*_25_=–1.93; *P*_75_=–0.47 superiority: 85/100 CV splits). In absolute terms, the final performance was about 8.2 yr MAE (*P*_25_=7.09; *P*_75_=9.19) and corresponded to an *R*^2^ score equivalent to 65% of explained variance (*P*_25_=0.58; *P*_75_=0.75; Supplementary Fig. S1).

**Fig. 4:**
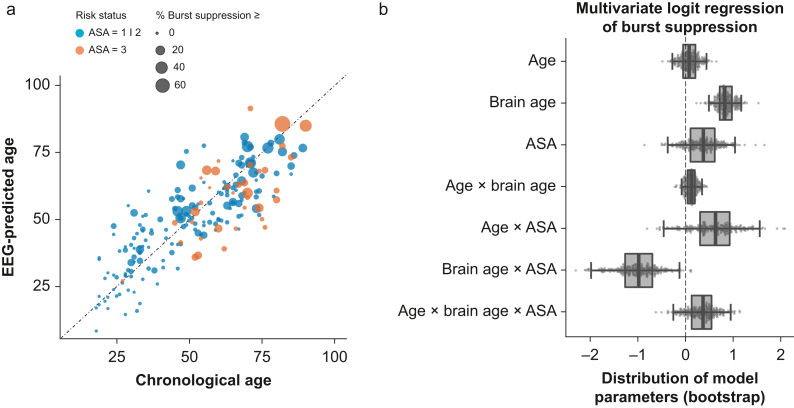
Clinical exploration of brain age derived from EEG during anaesthesia. (a) Plot of chronological age (x-axis) and brain age (y-axis) against the proportion of the EEG showing burst suppression (marker size). Binarised ASA score (ASA 1 or 2 *vs* ASA 3) is indicated by colour (*n*=204). (b) A logit-regression model summarising these trends. Box plots represent the two-sided 95% confidence interval (quantiles 2.5 and 97.5); individual dots indicate possible coefficients implied by the statistical uncertainty of the model. Brain age and the interaction with brain age showed the most consistent effects. Note that the exponential values of the coefficients can be interpreted as odds ratios of the proportion of burst suppression. Also note that in this statistical analysis, the brain-age coefficient reflects the age-prediction error of the machine learning model as, here, the effect of brain age is conditional on age, hence captures what is not explained by age.

For clinical exploration, we focused on the age-prediction signal under propofol anaesthesia in a subset of patients for whom burst suppression was annotated (DS5). We investigated the link between brain age, ASA score, and undesirable intraoperative burst suppression. Figure 4a plots model-based age predictions against chronological age and burst-suppression proportion for the previously analysed cohort of patients with good general health (DS3) and extrapolations to a distinct cohort of patients (DS4). Our results suggest more complex relationships between EEG-predicted age and burst suppression across patients of different ASA scores and chronological ages. One can observe that in younger ASA 1 or 2 patients, burst suppression was more frequent amongst those with higher brain age. This trend seems inverted in older ASA 3 patients, where bigger dots concentrate under the diagonal identity line. To formalise these relationships, we modelled the logit of the proportion of burst suppression as a weighted sum of scaled age, scaled brain age and ASA score, and their respective interaction terms (Fig. 4b). The analyses revealed a significant effect of brain age (β=0.85; standard error [se]=0.20; *t*[196]=4.4; *P*<0.0001). Given a standard deviation of brain age of about 16 yr, this suggests that across age and ASA score, burst suppression increased by 134% (exp[0.85]=2.34) for every 16 yr of brain age. Furthermore, a significant effect of brain age and ASA score emerged (β=–0.99; se=0.44; *t*[196]=–3.7; *P*=0.03), suggesting that the proportion of burst suppression was reduced by 63% (exp[–0.99]=37%) for every 16 yr of brain age in ASA 3 patients as compared with the other patients across all chronological ages. Of note, the term for brain age captures what is not explained by age (i.e. the error of machine learning model).^20^ The full model is reported in Supplementary Table S2.

**Fig. 5:**
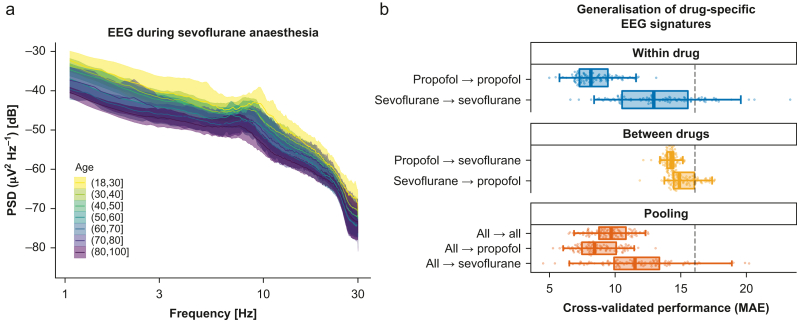
Divergence and pooling of EEG signatures by drug type. (a) The power spectral density (PSD) of EEG under sevoflurane anaesthesia (*n*=79; ASA ≤2). Visual conventions as in Figure 1a. Age appeared less consistently revealed by the PSD from sevoflurane as compared with propofol anaesthesia, raising the question if age prediction can be compared between these two drugs. (b) breakdown of modelling performance of the ‘spatial patterns’ model by drug type (100 Monte Carlo splits; conventions as in Fig 1b). Within-drug models were fitted and evaluated separately by drug type (blue). Between-drug models were fitted on one drug type but evaluated on the other drug type (orange). Pooling was attempted (dark orange) by combining data for fitting and evaluation on both drug types (first row). Separate scoring was performed by drug type (rows 2–3). Results suggest that performance was best when using EEGs from propofol anaesthesia. Whilst generalisation between drug types was poor, pooling EEGs from different drug types preserved the individual drug-specific performance. MAE, mean absolute error.

Visual inspection of EEG activity under sevoflurane anaesthesia (Fig. 5a; DS2) suggested higher EEG power levels and weaker correlation between EEG power and age. We formally contrasted these trends with the previous results through a linear mixed effects model regressing the log power (dB) on age, log frequency, drug type, and their interactions; intercepts varied by patient. Independently of frequency or drug type, EEG power declined by –0.10 dB; 95% CI [–0.13 to –0.08] for every year of age. Compared with propofol, sevoflurane led to 3.6 dB higher EEG power; 95% CI [1.21–6.10]. Notable interaction terms pointed at more complex EEG patterns. First, a two-way interaction suggested that differences between propofol and sevoflurane depended on frequency, implying that under sevoflurane anaesthesia, the power declined, on average, by about –1.00 dB more than under propofol anaesthesia per hertz; 95% CI [–1.27 to –0.76]. Second, a three-way interaction suggested that under sevoflurane anaesthesia, the effect of age may non-linearly increase log power by 0.01 dB Hz^−1^; 95% CI [<0.01–0.01]. The full model is reported in Supplementary Table S3.

Figure 5b presents comparisons between age-prediction models that were either separately fitted and evaluated within each drug type, fitted within one drug type and evaluated on the other drug type, or fitted and evaluated by pooling both drug types. When fitted separately, the prediction model from sevoflurane EEG performed about 4.9 yr of MAE (*P*_25_=2.7; *P*_75_=7.0) worse than propofol, inferior on 95/100 CV splits. Similarly, cross-drug generalisation of prediction models led to low prediction performance between 14 and 15 yr of MAE. By comparison, generalisation from propofol to sevoflurane EEG was by –1 yr of MAE more successful (*P*_25_=–1.8; *P*_75_=–0.25; superiority: 86/100 CV splits). Prediction models trained on the pooled EEG data from both drug types led to worse performance than the model trained under propofol EEG with about 1.3 yr higher MAE (*P*_25_=–0.31; *P*_75_=2.8; inferiority: 68/100 CV splits). However, results improved over the sevoflurane-based model by –3.7 yr of MAE (*P*_25_=–6.4; *P*_75_=–1.3; superiority: 85/100 CV splits). Considering the sub-scores of the pooled model for the observations under sevoflurane EEG suggested that combining both drugs led to an improvement of –1.6 yr of MAE (*P*_25_=–1.01; *P*_75_=4.0) compared with the model trained on sevoflurane EEG (superiority: 67/100 CV splits).

## Discussion

This study explored the feasibility of repurposing EEG monitoring data from general anaesthesia for building measures of brain ageing by importing machine learning approaches for brain-age prediction originally developed in the laboratory setting. Under propofol anaesthesia, using four electrodes only, we reached prediction performance comparable with reference studies using research-grade EEG^28 32^ with 20–62 electrodes. Model comparisons revealed that age-related information was present in spatial activity patterns distributed across the entire power spectrum. Clinical exploration of the propofol model highlighted important associations between brain-predicted age and the probability of burst suppression, which, however, depended on ASA score. Additional results suggested that the brain-age signature was specific to propofol and may not generalise to sevoflurane.

Clinical studies have identified a link between EEG power spectra in the alpha band (8–13 Hz), preoperative cognitive decline,^10^ intraoperative burst suppression,^33^^,^^34^ and postoperative cognitive disorders.^35^ This body of work pointed to the possibility that the EEG response to anaesthetic drugs may reveal the presence of neurodegenerative risk. Here, we extended this prior art by directly applying brain-age prediction models for EEG^28^ on a larger dataset of EEG obtained during anaesthesia. Under propofol anaesthesia, this approach has led to performance matching recent work with high-density MEG and EEG.^20^^,^^32^ Previous studies during anaesthesia have instead focused on EEG signatures closely related to anaesthesia monitoring, with particular emphasis on total power and alpha power.^6^^,^^36^ Our results have shown that the entire power spectrum and fine-grained correlations between signals collected at different electrodes may contain information relevant to developing biomarkers beyond anaesthesia monitoring.

However, prediction models constructed from EEG under sevoflurane anaesthesia were far less convincing and did not combine well with EEG collected under propofol anaesthesia. This may be intrinsically related to differences between the drugs regarding the mechanism of action. Propofol selectively activates gamma-aminobutyric acid Type A (GABA_A_) receptors,^37^ whereas sevoflurane acts on several synaptic pathways, potentially increasing the complexity of the signal across age.^38^ An alternative explanation might be that the dose of sevoflurane was more consistent between patients, as it relied on standard minimum alveolar concentration target values, and its elimination is less dependent on liver metabolism compared with propofol. Propofol dose was determined by a personalised TCI target value, chosen to stabilise general anaesthesia (i.e. to keep SEF95 in 8–13 Hz), potentially leading to higher inter-patient variability. This could, in principle, introduce confounding effects if, for example, the propofol requirement for stable anaesthesia depends on age and health. Higher propofol dose and brain age both dampen EEG power, whilst patients at risk of developing burst suppression might receive lower doses of propofol. In turn, this should lead to reduced propofol-induced dampening, obscuring the effect of brain age on EEG power. A similar effect may be present in the level of analgesia induced by the opioid. Patients undergoing the more painful orthopaedic surgery are generally older and more frail than those undergoing neuroradiology interventions, so have a higher chance of higher brain age. These patients received a higher dose of opioid (sufentanil in the case of our study) generally resulting in notable increase in EEG power in the delta and theta bands.^39^ Thus, patients at risk of developing burst suppression might receive higher doses of opioid, which boosts EEG power, which could make their brains ‘look younger’. That would be in line with observed changes of direction of association between brain age and burst suppression when comparing patients with lower (1or 2) *vs* higher (3) ASA scores, for whom higher brain age was associated with less burst suppression. Unfortunately, this hypothesis cannot be readily disambiguated using the present study. The critical pieces of information for moment-by-moment deconfounding were not captured: neither the dose of opioids, the TCI parameters controlling propofol dose, nor the changes in haemodynamic variables and noxious stimuli.^40^ Tackling these potential confounders calls for more complete data collection, ideally implemented with randomised controlled designs.

The present study has successfully applied concepts and methods from laboratory research in cognitive neuroscience^19^^,^^22^ on EEG monitoring data collected during general anaesthesia. Therein, our systematic model comparisons of EEG signatures, drug types, and patient populations have extended the scope of brain-age research to clinical real-world EEG collected in the absence of consciousness. Our findings motivate future research in neuroscience and anaesthesiology beyond monitoring of anaesthesia depth. These strengths of our work must be put in perspective with several limitations.

First, this proof-of-concept study does not validate brain age as a preoperative or postoperative biomarker of brain function, such as attention, memory, or dysfunction (*e.g.* delirium). Future work will have to close this gap through dedicated studies. The second limitation concerns the inconsistent findings regarding the variability of brain-age effects across drug types. Despite successful age-prediction results, the current work therefore does not yet present a ready-to-use brain-age measure as, for example, the MRI-based brain-age delta.^19^ To push this exploratory effort to the next level, future studies with ideally larger samples must focus on precise control and measurement of drug dose at any moment during anaesthesia. Preferably, propofol should be administered at a consistent dose to rule out confounding by clinical factors. Validation against pre-surgical brain-age estimates derived from gold-standard anatomical MRI^23^ or research-grade high-density EEG^28^ will be essential. Finally, future work should strive towards generalising the approach to patient populations beyond orthopaedic and neuroradiological surgery.

In conclusion, the present study points out the general feasibility of repurposing EEG from anaesthesia for learning biomarkers of brain ageing and health beyond the imminent perimeter of patient monitoring, complementing medical images and other biomarker modalities. To unleash this dormant potential of EEG monitoring data for clinical and public health research, collecting larger datasets with precisely documented drug dose, haemodynamic changes, and noxious stimuli will be key enabling factors.

## Authors’ contributions

Conceptualisation: AM, AG, DAE, EG, FV.

Data curation: CT, DS, JJ, FV.

Software: AG, DAE, DS, JC.

Formal analysis: AG, DAE, DS, JC.

Supervision: AG, DAE, EG.

Funding acquisition: DAE, EG.

Validation: AM, CT, DAE, DS, JC.

Investigation: DAE, DS.

Visualisation: DAE.

Methodology: DAE, DS.

Project administration: DAE.

Writing of original draft: DAE, DS.

Writing/review/editing: all authors.

## Funding

This work was supported by a 2018 ‘médecine numérique’ (for digital medicine) thesis grant issued by the Institut National de la Santé et de la Recherche Médicale (Inserm; French National Institute of Health and Medical Research) and the Institut National de Recherche en Sciences et Technologies du Numérique (Inria; French National Research Institute for the Digital Sciences). It was also supported by the Inserm–Inria artificial intelligence (AI) chair EDS-PeriOP and the ANR BrAIN AI chair (ANR-20-CHIA-0016).
