# Supplementary material for: Repurposing electroencephalogram monitoring of general anaesthesia for building biomarkers of brain ageing: an exploratory study
Source: BJA Open. 2023 Jun 16;7:100145. doi: 10.1016/j.bjao.2023.100145 (PMC10457469; doi:10.1016/j.bjao.2023.100145)
Supplement: Multimedia component 1 [file mmc1.pdf]

SUPPLEMENTARY MATERIAL

Supplementary Methods

EEG processing and feature engineering

We anonymized the monitoring EEG and converted it into the BIDS format[1] using the MNE-BIDS package[2]. We epoched the signal of each patient using 60-second sliding windows (10 seconds shift) using the MNE-Python software[3] and the MNE-BIDS pipeline[1] For every epoch we computed two different types of power-spectral features using the coffeine package[2]. We estimated the power-spectral density (PSD) using Welch's method and Hamming windows of 8 seconds (4 seconds shift), which can reveal EEG signatures commonly used in anaesthetic monitoring and clinical research. PSDs were estimated in 244 frequency bins between 0 and 30 Hz, and Hamming windows were averaged using a trimmed mean (cut = 25%) to increase robustness to artefacts. To explore the importance of spatio-spectral patterns, we computed the covariance between all 4 electrodes in 5 frequency bands adapted from reference publications on brain-age prediction[4, 5].

Construction of prediction models, model comparisons and statistical inference

The spatial-patterns model concatenated the band-specific EEG covariances after vectorization with Riemannian embeddings that accounted for non-linearities caused by EEG-volume conduction[6].
As in previous reference models[4, 7, 8], ridge regression[9] was used as the prediction algorithm. Its regularisation parameter was tuned by generalised cross validation[10] on a logarithmic grid of 100 values between [10^{-10}, 10^{100}] on each training set. For fair comparisons between models based on different numbers of features, the stacking method[11] was used as in previous brain-age publications[4, 12]. As the sample size was limited, instead of the non-linear random forest algorithm, we also used ridge regression in the stacking layer.
To estimate the expected generalisation performance, Monte Carlo cross validation (CV) with 100 splits and 20% testing data was applied with fixed random seeds supporting pairwise model comparisons.

Detection of burst suppression

To detect iso-electrical suppression (first part of the burst suppression pattern) from intraoperative EEG we adapted the method of a recent publication[13]. For each EEG, a trained clinician identified intra-operative periods based on the alpha-band. From this intraoperative EEG signal, we discarded flat artefacts searching for segments below 0.1 µV and high-amplitude epochs above 80 µV lasting at least 1 second. After the smoothing using a 30-seconds rolling average, we collected regions below 2.5 μV amplitude, then we sequentially applied a 0.2 second erosion, a 1 second dilation and an 0.8 second erosion. From this output we estimated the fraction of time spent in burst suppression during induction (the first 25 min) and maintenance periods (after 25 min). We focused on the maintenance period for the better statistical properties of the signal (more samples due to a longer period, less artefacts) and potentially lower false positive rate (drug-induced burst suppression can arise at induction more often). Burst suppression events were automatically excluded from analysis of EEG via artefact rejection. Annotations were not provided for 6 recordings (ds3 - ds5 in **Table 2**).

[1] https://mne.tools/mne-bids-pipeline/
[2] https://github.com/coffeine-labs/coffeine

Statistical analysis

To investigate correlations between age and the power spectrum, we computed linear mixed effect models using the lm4 package in R[14]. Statistical inference was obtained using confidence intervals and p-values approximated from the t-statistic as implemented by the sjPlot package (https://cran.r-project.org/web/packages/sjPlot/index.html). To investigate the associations between age, brain age and burst suppression across clinical groups, ordinary least squares regression was used on logit-transformed burst-suppression proportions as outcome. This approach allowed us to gauge complementarity of brain age and age in one modelling step[7, 15]. Next, to obtain parameter estimates and p-values, we reported confidence intervals based on the parametric bootstrap implemented in the arm package[16] as in a previous publication[15].

Supplementary Figures

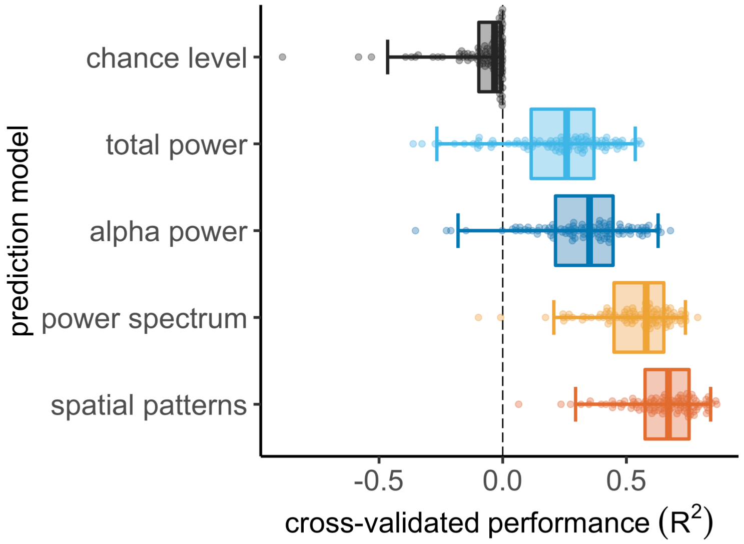

Fig. S1: Model comparisons using variance explained (R^2) as error metric. Same model and visual conventions as in **Fig 3B**.

Table S1. Linear Mixed Effect model of EEG log power as a function of frequency and age.

	psd		
Predictors	*Estimates*	*CI*	*p*
(Intercept)	-30.51	-31.80 – -29.22	**<0.001**
freqs [log]	-8.62	-8.77 – -8.48	**<0.001**
age	-0.10	-0.13 – -0.08	**<0.001**
freqs [log] * age	0.00	-0.00 – 0.00	0.286
**Random Effects**			
σ^2	25.73		
$\tau_{00\ participant\_id}$	8.03		
$N_{participant\_id}$	166		
Observations	42496		

Table S2. Generalised linear model of logit proportion of burst suppression as a function of age and brain age and risk status captured by the ASA score (sc = scaled).

	**qlogis(y)**		
Predictors	*Estimates*	*CI*	*p*
(Intercept)	-4.31	-4.59 – -4.04	**<0.001**
age sc	0.07	-0.30 – 0.44	0.718
brain age sc	0.86	0.47 – 1.25	**<0.001**
risk [high risk]	0.33	-0.35 – 1.01	0.340
age sc * brain age sc	0.13	-0.10 – 0.35	0.265
age sc * risk [high risk]	0.58	-0.31 – 1.47	0.200
brain age sc * risk [high risk]	-0.99	-1.85 – -0.13	**0.025**
(age sc * brain age sc) * risk [high risk]	0.34	-0.25 – 0.93	0.254
Observations	204		
R^2 / R^2 adjusted	0.349 / 0.326		

Table S3. Linear Mixed Effect model of EEG log power as a function of frequency, age and drug type.

	psd		
Predictors	*Estimates*	*CI*	*p*
(Intercept)	-30.51	-31.87 – -29.16	**<0.001**
freqs [log]	-8.62	-8.77 – -8.48	**<0.001**
age	-0.10	-0.13 – -0.08	**<0.001**
drug type [sevoflurane]	3.65	1.21 – 6.10	**0.003**
freqs [log] * age	0.00	-0.00 – 0.00	0.286
freqs [log] * drug type [sevoflurane]	-1.02	-1.27 – -0.76	**<0.001**
age * drug type [sevoflurane]	-0.01	-0.06 – 0.03	0.591
(freqs [log] * age) * drug type [sevoflurane]	0.01	0.00 – 0.01	**0.002**
**Random Effects**			
σ^2	25.69		
$\tau_{00\ participant\_id}$	8.96		
$N_{participant\_id}$	244		
Observations	62464		

References

1. Pernet CR, Appelhoff S, Gorgolewski KJ, et al. EEG-BIDS, an extension to the brain imaging data structure for electroencephalography. *Scientific data* Nature Publishing Group; 2019; **6**: 1–5

2. Appelhoff S, Sanderson M, Brooks TL, et al. MNE-BIDS: Organizing electrophysiological data into the BIDS format and facilitating their analysis. *The Journal of Open Source Software* [Internet] 2019; **4** Available from: https://mne.tools/mne-bids/

3. Gramfort A, Luessi M, Larson E, et al. MNE software for processing MEG and EEG data. *Neuroimage* 2014; **86**: 446–60

4. Sabbagh D, Ablin P, Varoquaux G, Gramfort A, Engemann DA. Predictive regression modeling with MEG/EEG: from source power to signals and cognitive states. *Neuroimage* 2020; **222**: 116893

5. Engemann DA, Mellot A, Höchenberger R, et al. A reusable benchmark of brain-age prediction from M/EEG resting-state signals [Internet]. bioRxiv. 2021 [cited 2022 Jan 25]. p. 2021.12.14.472691 Available from: https://www.biorxiv.org/content/biorxiv/early/2021/12/16/2021.12.14.472691

6. Congedo M, Barachant A, Bhatia R. Riemannian geometry for EEG-based brain-computer interfaces; a primer and a review. *Brain-Computer Interfaces* Taylor & Francis; 2017; **4**: 155–74

7. Engemann DA, Kozynets O, Sabbagh D, et al. Combining magnetoencephalography with magnetic resonance imaging enhances learning of surrogate-biomarkers. *Elife* eLife Sciences Publications Limited; 2020; **9**: e54055

8. Engemann DA, Mellot A, Höchenberger R, et al. A reusable benchmark of brain-age prediction from M/EEG resting-state signals. *Neuroimage* Elsevier BV; 2022; **262**: 119521

9. Hoerl AE, Kennard RW. Ridge regression: Biased estimation for nonorthogonal problems. *Technometrics* Taylor & Francis Group; 1970; **12**: 55–67

10. Golub GH, Heath M, Wahba G. Generalized Cross-Validation as a Method for Choosing a Good Ridge Parameter. *Technometrics* [Taylor & Francis, Ltd., American Statistical Association, American Society for Quality]; 1979; **21**: 215–23

11. Wolpert DH. Stacked generalization. *Neural Netw* Elsevier; 1992; **5**: 241–59

12. Liem F, Varoquaux G, Kynast J, et al. Predicting brain-age from multimodal imaging data captures cognitive impairment. *Neuroimage* 2017; **148**: 179–88

13. Cartailler J, Parutto P, Touchard C, Vallée F, Holcman D. Alpha rhythm collapse predicts iso-electric suppressions during anesthesia. *Communications biology* Nature Publishing Group; 2019; **2**: 1–10

14. Bates D, Sarkar D, Bates MD, Matrix L. The lme4 package. *R package version* 2007; **2**: 74

15. Dadi K, Varoquaux G, Houenou J, Bzdok D, Thirion B, Engemann D. Population modeling with machine learning can enhance measures of mental health. *Gigascience* Oxford Univeristy Press; 2021; **10**: 1–16

16. Gelman A, Hill J. Data analysis using regression and multilevel/hierarchical models. Cambridge University Press; 2007.
